# Motivational Strategies in Digital Interventions for Children With Attention-Deficit/Hyperactivity Disorder (ADHD): A Scoping Review

**DOI:** 10.7759/cureus.117722

**Published:** 2026-10-10

**Authors:** Nailah Ali, Shreehari Shreedhar, Patricia Oconnell, Louise Lambert

**Affiliations:** 1 Public Health, Bjørnholt Upper Secondary School, Oslo, NOR; 2 Public Health, Yasmina British Academy, Abu Dhabi, ARE; 3 College of Medicine, Brighton and Sussex Medical School, Brighton, GBR; 4 Psychology, Happiness Matters, Dubai, ARE

**Keywords:** attention-deficit/hyperactivity disorder (adhd), digital interventions, executive functioning, motivational strategies, public health

## Abstract

Attention-deficit/hyperactivity disorder (ADHD) is associated with impairments in executive functioning and difficulties sustaining attention and goal-directed behaviour. Digital interventions offer a promising avenue for addressing these difficulties because they can modulate task demands and deliver immediate reinforcement, although evidence for their superiority over conventional interventions remains limited. This scoping review mapped the motivational strategies, defined as discrete intervention features intended to encourage engagement, participation, persistence, or responsiveness, distinct from the underlying cognitive training itself, incorporated into digital interventions targeting executive functioning and ADHD-related outcomes in children aged 6-12 years. Six studies published between 2018 and 2026, identified through a PubMed search, met the inclusion criteria. Five were randomized controlled trials, and one was a pilot study. All six studies reported immediate feedback, five reported adaptive difficulty, four reported points or scores, and three reported in-game rewards and progress tracking. Each of virtual currency, narrative, customization, and explicit positive reinforcement was reported in one of six studies. Competitive leaderboards were reported in none. Although all six studies reported improvement in at least one outcome, heterogeneity in intervention design and outcome assessment, together with the very limited evidence base and the single-database search, precluded attribution of these findings to specific motivational strategies. It remains a hypothesis, rather than an established finding, that motivational design may influence how children interpret performance, regulate effort, and persist when faced with cognitive demand. Future research should examine motivational strategies as distinct components of intervention design and determine whether particular strategies are associated with sustained engagement and improvements in executive functioning or ADHD-related outcomes.

## Introduction and background

Attention-deficit/hyperactivity disorder (ADHD) is one of the most common neurodevelopmental disorders in childhood, with a prevalence of more than 5% among children and adolescents worldwide [1]. ADHD is characterised by persistent patterns of inattention, hyperactivity, and impulsivity, and is frequently accompanied by impairments in executive functions such as working memory, inhibitory control, and cognitive flexibility. ADHD is diagnosed more frequently in boys than in girls [1]. Reported male-to-female ratios have declined over time, from 7.5:1 to 3:1 at early school age and from 8.1:1 to 1.6:1 in adolescents, which may reflect improved recognition of ADHD in girls [1]. Boys are more frequently diagnosed and tend to show higher levels of hyperactive-impulsive behaviour, whereas girls more often present with inattentive symptoms. Symptoms persist into adulthood in a substantial proportion of cases [1]. Despite the extensive body of research devoted to ADHD, with approximately 20,000 publications indexed in PubMed during the decade preceding 2020, the field still lacks a clear understanding of which intervention components drive engagement and symptom improvement [1]. The central problem is that many children with ADHD do not receive effective, sustained intervention, and the components that drive engagement remain poorly understood. Executive functions comprise higher-order cognitive processes that support the regulation and coordination of goal-directed behaviour, including the ability to maintain and manipulate information, inhibit prepotent responses, and flexibly adapt behaviour. These impairments may extend into academic, social, and everyday functioning, making the development of effective and sustainable interventions particularly pertinent. Children with ADHD, their families, clinicians, and intervention developers would benefit from clearer identification of which motivational components are actually being used, so that future interventions can be designed with greater precision and reporting can become more replicable. A scoping review was therefore chosen because the aim was to map the types, frequency, implementation, and reporting of motivational strategies across a heterogeneous evidence base, rather than to estimate a pooled effect.

Digital interventions, such as exergaming platforms, tablet-based cognitive training applications, and video-game-like digital therapeutics, represent a promising approach to the treatment of executive function difficulties in children with ADHD [2]. Their capacity to allow for repeated and individualised training is particularly relevant to a disorder in which sustained participation may itself prove difficult. Yet the efficacy of an intervention cannot be considered separately from the conditions under which a child continues to engage with it, and adherence to digital interventions is itself variable [2]. Motivation, in this context, refers to the psychological processes that initiate, sustain, and direct goal-directed behaviour. Motivational strategies were defined as game-design elements incorporated into a non-game intervention to encourage engagement, adherence, or effort. These were classified using the framework proposed by Deterding et al., which distinguishes feedback, points, rewards, adaptive difficulty, progression, narrative, customisation, and competitive elements [3]. This framework was chosen because it is the most widely cited operationalisation of gamification in digital health research, and because it permits clearer separation of motivational design features from cognitive training components that may share similar surface characteristics. These may include immediate feedback, points, rewards, adaptive difficulty, and progress tracking. Some of these features constitute gamification, broadly understood as the incorporation of game-like elements into a non-game intervention or task to increase engagement, such as points, rewards, progression, challenges, or narrative elements [3]. In ADHD, altered reward processing and heightened sensitivity to immediate reinforcement may affect how children respond to motivational features. Children may disengage more quickly when reinforcement is delayed, which makes immediate feedback and reward-based elements particularly relevant to intervention design [4]. Motivation may constitute more than a peripheral feature of intervention design. It may be one of the conditions upon which continued engagement depends.

Existing evidence, however, has tended to entertain the question of whether digital and game-based interventions are effective rather than what their constituent motivational strategies actually are. Cortese et al. reported that cognitive training improved working memory but produced limited effects on ADHD symptoms when outcomes were assessed by blinded raters [5]. Mao et al. more recently reported a moderate overall effect on cognitive performance following game-based interventions, albeit with substantial heterogeneity across studies [6]. These findings underscore the complexity of evaluating digital and game-based approaches. They do not, however, elucidate which components of such interventions may contribute to engagement or treatment response.

Research gap

Although digital interventions for ADHD have received increasing attention, existing systematic reviews have predominantly examined efficacy rather than the motivational strategies incorporated within these interventions [5,6]. Cortese et al. demonstrated that cognitive training may improve specific neuropsychological outcomes without producing corresponding improvements in ADHD symptoms [5]. Mao et al. subsequently reported moderate effects of game-based interventions on cognitive performance, albeit alongside substantial heterogeneity, leaving the contribution of individual game components uncertain [6]. Consequently, although motivational strategies are frequently described in digital interventions for ADHD, their types, frequency, implementation, and reporting have not been systematically mapped. It remains unclear which strategies are most commonly used and how they are described. The present review therefore maps these components rather than attempting to determine their effectiveness. A scoping review was chosen because it permits systematic mapping of a heterogeneous and developing evidence base, rather than pooled estimation of intervention effects.

Objectives

In accordance with the Preferred Reporting Items for Systematic Reviews and Meta-Analyses extension for Scoping Reviews (PRISMA-ScR) guidelines [7], this scoping review sought to map the motivational strategies employed within digital interventions targeting executive functioning in children with ADHD. A scoping review was chosen because the aim was to map the range and reporting of motivational strategies rather than to estimate their pooled effect, and because the heterogeneity of intervention designs and outcome measures would preclude meaningful meta-analysis. The research question, framed using the Population, Concept, Context (PCC) structure, was: What motivational strategies are incorporated into digital interventions for children aged 6 to 12 years with ADHD, how are they implemented and reported, and what gaps remain in the evidence?

The objectives of this review were to identify the range of motivational strategies used in digital interventions for children with ADHD, to describe how these strategies were implemented and reported, and to examine methodological characteristics that affect interpretation of the evidence. Such synthesis is particularly pertinent to the development of interventions wherein engagement is regarded not as a mere accompaniment to treatment, but as a potentially consequential component of intervention design.

## Review

Methods

Study Design

The scoping review sought to map the existing data and clinical evidence examining motivational strategies in digital interventions for children and adolescents with ADHD. Given substantial heterogeneity among studies with respect to intervention types, motivational strategies, outcome measures, and study designs, findings were synthesised descriptively. The search was conducted in PubMed in March, 2026. No protocol was registered for this review.

Search Strategy

A literature search was conducted in PubMed to identify relevant studies. The search strategy combined Medical Subject Headings (MeSH) terms with title/abstract keywords using Boolean operators (OR and AND). Terms relating to ADHD included the MeSH term “Attention Deficit Disorder with Hyperactivity” and the keyword “ADHD.” Terms relating to digital and game-based interventions included the MeSH term “Video Games” and title/abstract keywords including “Serious Games,” “exergam*,” “digital therapeutic*,” “computerized training,” “gamif*,” “reward*,” and “token economy.” Terms relating to executive functioning included the MeSH term “Executive Function” and title/abstract keywords including “working memory,” “cognitive control,” and “inhibition.” Terms within each concept were combined using OR, and the resulting concept groups were combined using AND. The search was restricted to human studies involving children aged 6-12 years and publications from 2000 to 2026.

Study Selection and Screening

Data extraction was guided by the PCC framework and the Joanna Briggs Institute (JBI) guidance for scoping reviews, and was done by two reviewers. Variables screened were author, year, country, study design, sample size, age, gender distribution, ADHD diagnostic criteria, intervention characteristics, motivational strategies, assessment tools, outcomes, effect sizes, covariates, and reported limitations. The study selection process was conducted in two stages. First, titles and abstracts of all records identified through the PubMed search were independently screened by two reviewers. Disagreements were resolved through discussion. Second, potentially relevant articles underwent full-text review to determine eligibility. The study selection process was documented using a PRISMA flow diagram illustrating the identification, screening, eligibility assessment, and inclusion of studies (Figure 1). Studies were included if they investigated digital interventions incorporating motivational strategies in children aged 6-12 years with ADHD and assessed executive function or ADHD-related outcomes. No geographical restrictions were applied. Eligible study designs included randomised controlled trials and pilot studies. Only studies involving human participants were considered. Articles were restricted to English-language publications and were required to be available as full free text. Studies were excluded if they did not involve children aged 6-12 years with ADHD, did not investigate a digital intervention incorporating motivational strategies, did not assess executive function or ADHD-related outcomes, were not randomised controlled trials or pilot studies, did not involve human participants, were not published in English, or were not available as full free text. Screening was conducted manually, with records managed in Microsoft Excel (Microsoft Corp., Redmond, WA, USA).

**Figure 1:**
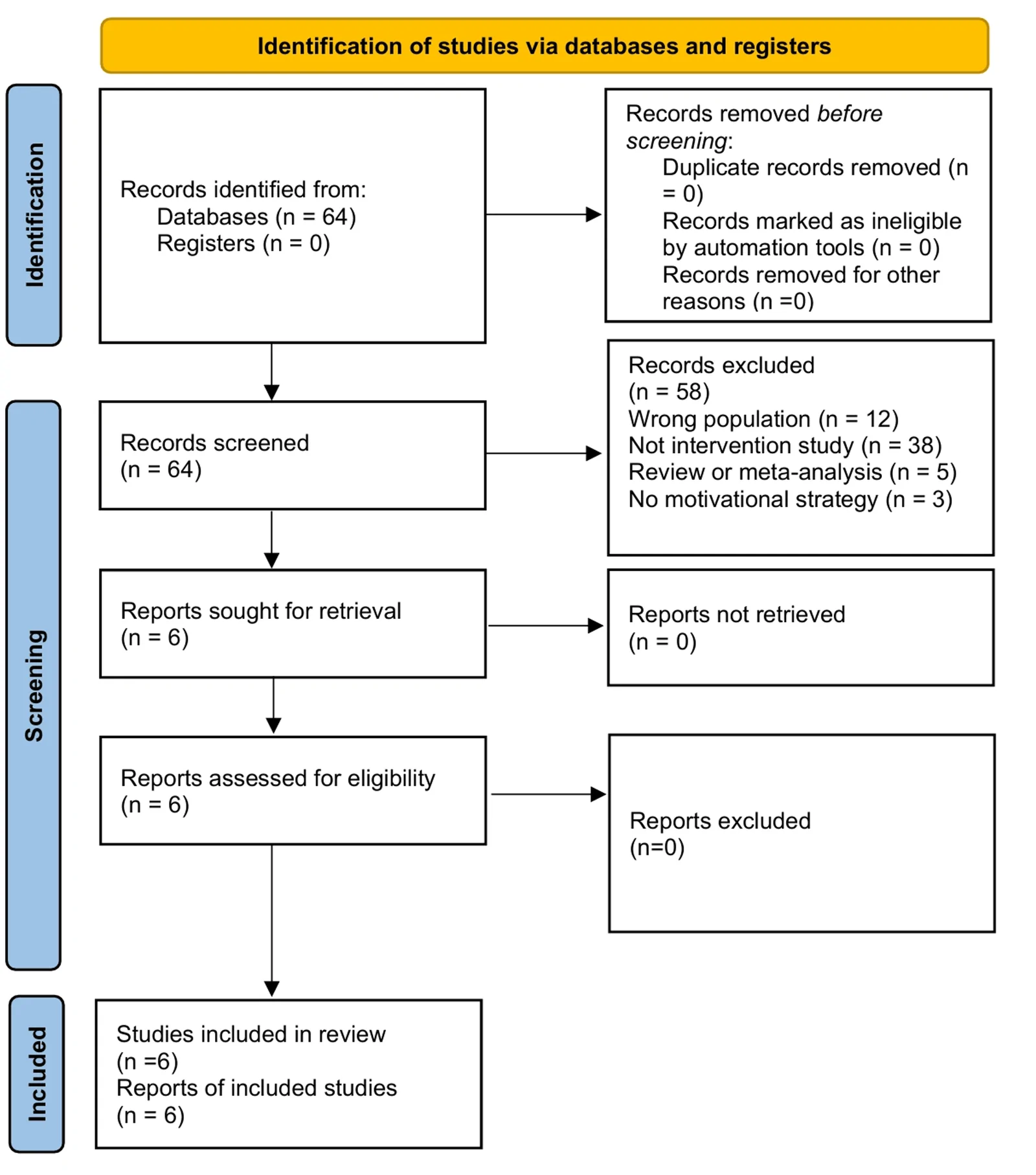
PRISMA flow diagram of the study selection process, showing records identified, screened, excluded, and included in the review.

Risk of Bias Assessment

Methodological quality of the included studies was assessed using the relevant JBI critical appraisal tools [8,9] presented in Figure 2. Two reviewers independently assessed each study using the JBI checklists. "Yes" responses were rated as low risk, "Unclear" as some concerns, and "No" as high risk. Randomised controlled trials were assessed using the JBI critical appraisal checklist for randomised controlled trials, with assessment focusing on randomisation, allocation concealment, blinding of participants and personnel, blinding of outcome assessors, completeness of outcome data, and selective reporting. Blinding was largely absent across the trials. The pilot single-arm study (Jung et al., 2025) [10] was assessed using the JBI critical appraisal checklist for quasi-experimental studies. The cause-and-effect relationship was clear, multiple pre-post measurements were taken, and outcomes were measured consistently and reliably. However, no control group was present, and follow-up completeness was unclear. The results of the critical appraisal were used to assess the methodological strengths and limitations of the included evidence and to inform interpretation of the findings.

**Figure 2:**
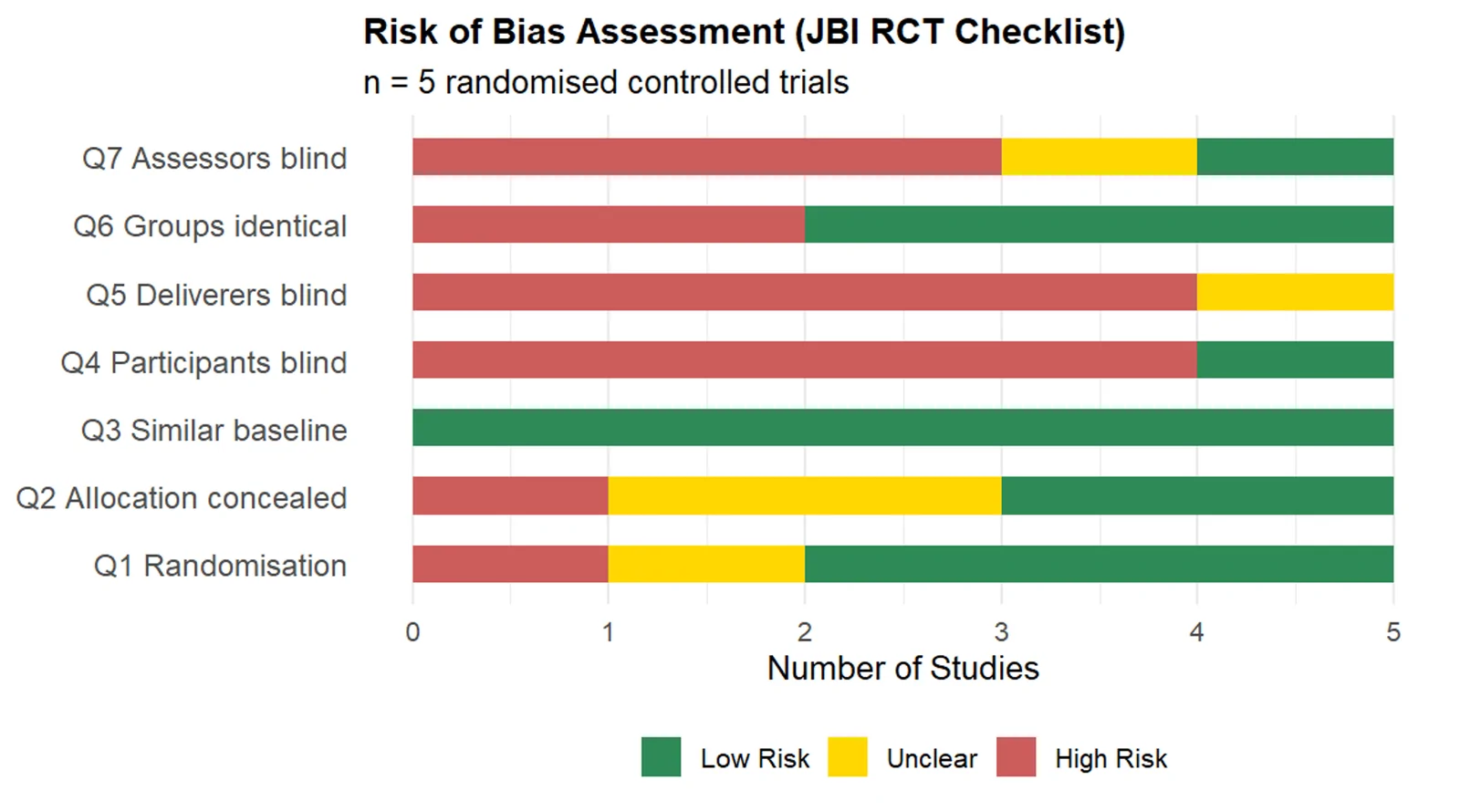
Risk of bias assessment of the five RCTs using the JBI critical appraisal checklist for RCTs. JBI: Joanna Briggs Institute; RCTs: randomised controlled trials

Results

Geographical and Population Study Areas

Table 1 presents the characteristics and participant demographics of the included studies. A total of six studies met the inclusion criteria and were included in the scoping review. All studies were published between 2018 and 2026. Studies were conducted in Switzerland [11,12], China [13,14], South Korea [10], and the USA [2]. All studies were conducted in high-income or upper-middle-income countries, with no studies identified from low-income or lower-middle-income countries.

**Table 1:** Study characteristics and demographic. ADHD: attention-deficit/hyperactivity disorder; RCT: randomised controlled trial; ICD-10: International Classification of Diseases, 10th Revision; DSM: Diagnostic and Statistical Manual of Mental Disorders, Fifth Edition

Author (year)	Country	Design	N analysed	Age range	Gender distribution	ADHD diagnostic criteria	Comorbidities/medication
Benzing et al. (2018) [12]	Switzerland	RCT	46	6-12 years	82.6% male, 17.4% female	ICD-10	N/A
Benzing and Schmidt (2019) [11]	Switzerland	RCT	44	6-12 years	82.4% male, 17.6% female	ICD-10	Medicated and unmedicated
Zhao et al. (2024) [13]	China	RCT	80	6-12 years	78.8% male, 21.2% female	DSM-5	N/A
Kollins et al. (2020) [2]	USA	RCT	329	6-12 years	69.0% male, 31.0% female	DSM-5	Medication-free; comorbidities excluded
Jung et al. (2025) [10]	South Korea	Single-arm pilot, pre-post	19	6-12 years	73.7% male, 26.3% female	DSM-5	Medicated and unmedicated
Yan et al. (2026) [14]	China	RCT	74	6-12 years	≈78.0% male, ≈22.0% female	DSM-5	Medication-free; comorbidities excluded

Five studies were randomised controlled trials [2,11-14], while one was a single-arm pilot study with a pre-post design [10]. Analysed sample sizes ranged from 19 to 329 participants. All six studies included children aged 6-12 years with a confirmed diagnosis of ADHD. No studies exclusively included adolescents aged 13-18 years or children younger than six years.

All studies reported a male predominance, with female participants comprising 17.4%-31.0% of the analysed samples. ADHD diagnosis was established using the Diagnostic and Statistical Manual of Mental Disorders, Fifth Edition (DSM-5) criteria in four studies [2,10,13,14] and the International Classification of Diseases, 10th Revision (ICD-10) criteria in two studies [11,12]. Most studies excluded participants with severe psychiatric comorbidities. Two studies excluded participants taking ADHD medication [2,14], while other studies included both medicated and unmedicated participants [10,11,13]. IQ was assessed in several studies [2,13,14] with eligibility criteria typically requiring an IQ of ≥70 or ≥80.

Motivational Strategies

All six included studies incorporated at least one motivational strategy. Immediate feedback, delivered visually or auditorily, was reported in all six studies [2,10-14]. Table 2 presents the motivational strategies reported in the included studies. Zhao et al. (2024) used real-time performance feedback during augmented reality (AR) cognitive-physical training, with scores earned for correct and timely task completion and difficulty adjusted according to performance [13]. Benzing and Schmidt used an adaptive exergame in which players competed against their own high scores, with points awarded for precise movement and automatic difficulty adjustment to the player's proficiency [11]. Benzing et al. used a single session of the same exergame, with points awarded for movement and precision, although adaptive difficulty was not reported for the single-session format [12]. Kollins et al. [2] used a video game-like digital therapeutic with reward loops, unlocking of new environments, and real-time adaptive difficulty. Yan et al. (2026) [14] used a tablet-based attention training game with visual and auditory positive feedback when sustained attention was detected, and negative visual feedback when attention declined, alongside game-like rewarding effects of the avatar. Jung et al. (2025) [10] used a gamified multitasking mobile application with in-app rewards, virtual currency, village-building, character and vehicle customisation, narrative elements, and performance-based unlocking of content. Adaptive difficulty was reported in five studies [2,10,11,13,14], while points or scores were reported in four studies [2,10-12]. In-game rewards were reported in three studies [2,10,14], and progress tracking or levelling was reported in three studies [2,10,13]. Token economy or virtual currency, storyline or narrative, village-building or customisation was reported in one study [10], and explicit positive reinforcement was reported in one study [14]. No study reported the use of competitive leaderboards [2,10-14].

**Table 2:** Motivational strategies reported in included studies.

Motivational strategy	Number of studies	Studies (author, year)
Immediate feedback	6	Zhao et al. (2024) [13]; Benzing and Schmidt (2018, 2019) [11,12]; Kollins et al. (2020) [2]; Yan et al. (2026) [14]; Jung et al.(2025) [10]
Adaptive difficulty	5	Zhao et al. (2024) [13]; Benzing and Schmidt (2019) [11]; Kollins et al. (2020) [2]; Yan et al. (2026) [14]; Jung et al. (2025) [10]
Points or scores	4	Benzing and Schmidt (2018, 2019) [11,12]; Kollins et al. (2020) [2]; Jung et al. (2025) [10]
In-game rewards	3	Kollins et al. (2020) [2]; Yan et al. (2026) [14]; Jung et al. (2025) [10]
Progress tracking or levelling	3	Zhao et al.(2024) [13]; Kollins et al. (2020) [2]; Jung et al. (2025) [10]
Token economy or virtual currency	1	Jung et al. (2025) [10]
Storyline or narrative	1	Jung et al. (2025) [10]
Village-building or customisation	1	Jung et al. (2025) [10]
Explicit positive reinforcement	1	Yan et al. (2026) [14]
Competitive leaderboards	0	None reported

Assessment Tools and Key Findings

Table 3 presents the outcome measures and statistical characteristics of the included studies. Laboratory-based measures included the Test of Variables of Attention (TOVA), which assesses sustained attention and inhibitory control [15]; the Eriksen Flanker task, which assesses inhibitory control and interference suppression [16]; the Simon task, which assesses interference control and response inhibition [17]; the Stroop Color and Word Test, which assesses selective attention and processing speed [18]; the ADHD Diagnostic System (ADS), which assesses sustained attention and impulsivity [19]; the Digit Span subtest, which assesses verbal working memory [20]; and the Color Span Backwards task, which assesses visual-spatial working memory [21]. Parent-rated measures included the Swanson, Nolan, and Pelham Questionnaire (SNAP-IV), which assesses inattention, hyperactivity, and impulsivity [22]; the Behavior Rating Inventory of Executive Function (BRIEF), which assesses everyday executive function [23]; the Korean ADHD Rating Scale (K-ADHD-RS), which assesses ADHD symptom severity [24]; the Korean Conners' Parent Rating Scale (K-CPRS), which assesses behavioural and emotional difficulties [25]; and the Conners third edition (Conners-3), which assesses ADHD and related behaviours [26]. All six studies reported statistically significant improvements in at least one measured outcome, though the magnitude of these effects was non-uniform. The largest effect was observed for parent-rated ADHD symptoms, reported by Zhao et al. (2024) with a Cohen's d of 1.258 [13]. Benzing et al. (2018) reported eta-squared values of 0.117 for inhibition and 0.113 for switching [11,12]. Benzing and Schmidt (2019) reported Cohen's d values of 0.58 for inhibition and 0.65 for switching [11,12]. Kollins et al. (2020) reported a median change of 0.88 on the TOVA Attention Performance Index (API) but did not provide a traditional effect size, which limits direct comparability [2]. Yan et al. (2026) reported a moderate improvement in attention on the TOVA API, with an eta-squared of 0.091 [14]. Jung et al. (2025) reported moderate improvements in parent-rated ADHD symptoms and behaviour problems, with Cohen's d values of 0.69 on the K-ADHD-RS and 0.72 on the K-CPRS (10). A discernible pattern emerged in which parent-rated outcomes tended to confer larger effects than laboratory-based measures [2,10,13,14]. Whether this constitutes genuine intervention impact or arises from differences in measurement sensitivity remains unclear from the present evidence. The heterogeneity in effect sizes across studies is nevertheless a notable feature of this evidence base.

**Table 3:** Outcome measures and statistical characteristics of included studies. TOVA: Test of Variables of Attention; SNAP-IV: Swanson, Nolan, and Pelham Questionnaire; BRIEF: Behavior Rating Inventory of Executive Function; ADS: ADHD Diagnostic System; K-ADHD-RS: Korean ADHD Rating Scale; K-CPRS: Korean Conners' Parent Rating Scale; ANCOVA: analysis of covariance; CI: confidence interval

Author (year)	Assessment tools	Key reported outcome	Covariate adjustment	Key conclusions
Benzing et al. (2018) [12]	Flanker [16]; Color Span Backwards [21]; Conners-3 [26]	Inhibition: eta-squared = 0.117; Switching: eta-squared = 0.113	Pre-test values; ANCOVA	Acute exergaming improved reaction times for inhibition and switching, but not accuracy or working memory.
Benzing and Schmidt (2019) [11]	Flanker [16]; Simon [17]; Color Span Backwards [21]; Conners-3 [26]	Inhibition: Cohen’s d = 0.58; Switching: Cohen’s d = 0.65	Pre-test values; ANCOVA	Eight weeks of exergaming improved inhibition and switching reaction times, general psychopathology, and motor abilities.
Zhao et al. (2024) [13]	SNAP-IV [22]; BRIEF [23]	SNAP-IV Total: Cohen’s d = 1.258 (95% CI 0.804-1.712)	Age, sex, ADHD subtype, academic performance, sociability; multivariable regression + permutation test	BrainFit produced large improvements in parent-rated ADHD symptoms and moderate improvements in executive function.
Kollins et al. (2020) [2]	TOVA [15]; BRIEF [23]	TOVA API: median change = 0.88 (95% CI 0.24-1.49)	Age, sex, baseline TOVA, site, medication washout status; Wilcoxon rank-sum + sensitivity analyses	AKL-T01 improved objectively measured attention but not parent-rated symptom measures.
Yan et al. (2026) [14]	TOVA [15]; SNAP-IV [22]	TOVA API: eta-squared = 0.091	Baseline TOVA; ANCOVA	EEG neurofeedback enhanced attention training outcomes compared with the same game without neurofeedback.
Jung et al. (2025) [10]	Stroop [18]; ADS [19]; Digit Span [20]; K-ADHD-RS [24]; K-CPRS [25]; in-app performance	K-ADHD-RS: Cohen’s d = 0.69; K-CPRS: Cohen’s d = 0.72	None; paired t-test/Wilcoxon	StarRuckus improved parent-rated ADHD symptoms and behaviour problems in a single-arm pilot.

Statistical Methodology and Confounding Adjustment

Statistical methods varied across the included studies. Analysis of covariance (ANCOVA) was used in three studies to account for pre-test or baseline values [11,12,14]. Zhao et al. (2024) used multivariable regression with a permutation test [13], while Kollins et al. (2020) used the Wilcoxon rank-sum test with sensitivity analyses [2]. Jung et al. (2025) used paired t-tests and Wilcoxon tests without covariate adjustment [10].

Five of the six studies reported adjustment for one or more covariates. Benzing and Schmidt (2018, 2019) adjusted for pre-test values [11,12], while Yan et al. (2026) adjusted for baseline TOVA performance [14]. Zhao et al. (2024) adjusted for age, sex, ADHD subtype, academic performance, and sociability [13]. Kollins et al. (2020) adjusted for age, sex, baseline TOVA, study site, and medication washout status [2]. Jung et al. (2025) did not report covariate adjustment [10].

Author Reported Limitations

Several limitations were reported across the included studies [2,10,12,14]. Jung et al. (2025) reported a small sample size, lack of a control group, and unblinded assessments [10]. Zhao et al. (2024) reported limitations related to lack of blinding, expectancy effects among parents completing assessments, single-centre recruitment, and the inability to assess durability of treatment effects [13]. Several studies did not include follow-up assessments beyond the intervention period. Benzing and Schmidt (2019) reported 14% attrition [11], while Kollins et al. (2020) noted limited generalisability associated with medication-free samples, exclusion of comorbidities, and the selected IQ range [2].

Variation Across Included Studies

The included studies varied in intervention format, delivery platform, duration, session frequency, motivational strategies, assessment tools, and statistical methods. Intervention duration ranged from a single 15-minute session to eight weeks, while delivery formats included exergaming, AR-based cognitive-physical training, video game-like digital therapeutics, and gamified multitasking training. Outcome measures also varied across studies, including laboratory-based cognitive tasks, parent-rated questionnaires, and in-app performance measures. Analysed sample sizes ranged from 19 to 329 participants. The included studies also differed in study design, with five randomised controlled trials and one single-arm pilot study.

Recruitment Settings

Recruitment settings varied across the included studies. Zhao et al. (2024) recruited participants through an ADHD outpatient clinic [13]. Yan et al. (2026) recruited participants through a Children's Hospital outpatient clinic [14]. Benzing and Schmidt (2018, 2019) recruited participants through parent and caregiver associations [11,1]. Kollins et al. (2020) recruited participants across 20 research sites [2], while Jung et al. (2025) recruited participants through a community child centre and an online community [10].

Discussion

Key Findings in Existing Literature

This scoping review sought to map the motivational strategies incorporated into digital interventions targeting executive functioning and ADHD-related outcomes in children with ADHD. Six studies met inclusion criteria, all published between 2018 and 2026, and all conducted within high-income or upper-middle-income settings. Each incorporated at least one motivational strategy. The resulting catalogue is preliminary and should be read as such rather than as a comprehensive account of the field.

These findings are consistent with previous reviews of digital and game-based interventions for ADHD, which have reported improvements in selected cognitive outcomes alongside considerable heterogeneity [5,6]. The present review extends that literature by changing the question from whether digital interventions may be efficacious to what, precisely, is being incorporated into them to prompt participation and persistence.

Cortese et al. reported that cognitive training improved working memory but produced limited effects on ADHD symptoms when outcomes were assessed by blinded raters [5]. In their meta-analysis of 16 randomised controlled trials, working memory improved with standardised mean differences of 0.52 for verbal and 0.47 for visual working memory, yet effects on ADHD symptoms were very small when assessed by blinded raters, at 0.20 for total symptoms [5]. Mao et al. reported a moderate overall effect on cognitive performance following game-based interventions, with substantial heterogeneity across studies [6]. In a meta-analysis of 20 randomised controlled trials with 1,376 participants, the overall effect size was 0.42, with heterogeneity of 86.6 percent; effects were strongest for attention, at 0.724, and weaker for working memory, at 0.486, and inhibitory control, which was not statistically significant [6]. Neither review examined the motivational components of the interventions they pooled. Mao et al. found high heterogeneity. This review found the same problem for a different reason. The studies use different strategies and measure and report them differently. Lee et al. (2025) conducted a systematic review and meta-analysis of eight randomised controlled trials synthesizing game-based digital interventions using the notion of experiential learning theory [27]. This review was identified during data screening and excluded from the present review because it is a review rather than a primary study, but it is cited here for comparison. Lee et al. reported significant improvements in visuospatial short-term memory and visuospatial working memory but no significant effects on behavioural domains such as inhibition or monitoring. Lee et al. listed the gaming elements they found, such as rewards, points, badges, adaptive difficulty, and narrative, but they did not separate these elements from each other or test which ones mattered [27]. The present review therefore extends their work by mapping which specific strategies were used across studies and how they were reported, rather than estimating their combined effect. Both reviews converge on a similar limitation, namely that motivational components are frequently bundled within the same intervention, which prevents attribution of outcomes to any single component.

Competition constitutes, in game design generally, one of the more common motivational tools [3], and its absence from all six included studies as well as the aforementioned reviews by Cortese et al., Mao et al., and Lee et al. is of interest [5,6,27]. Nonetheless, no single interpretation can be discerned. It may be that researchers avoided it deliberately, given that frustration thresholds in children with ADHD are often lower [4]. It may be that the constraints of trial design rendered competition impracticable. It may be that the omission was never deliberated at all. Whether the omission was deliberate, practical, or accidental, the six studies and the reviews that preceded them provide no material by which the question might be settled.

Motivational Strategies in Digital Interventions

The strategies identified in this review are not interchangeable. Immediate feedback and adaptive difficulty both respond to performance, but they do so differently. Feedback returns information about what the child has just done, whereas adaptive difficulty changes what the child will do next. Points and scores mark progress symbolically, while rewards and token economies attach exchange value that can be accumulated. Narrative frames the intervention thematically, customisation grants ownership, and explicit positive reinforcement carries social weight. Treating these as one feature, as the term gamification often does, would remove the distinctions this mapping was intended to reveal [3].

Jung et al. (2025) showed this complexity particularly well, incorporating virtual currency, narrative elements, and customisation within the same intervention, whilst Yan et al. (2026) explicitly reported positive reinforcement [10,14]. Such differences suggest that motivational design is not uniform, but rather consists of overlapping strategies frequently combined within individual interventions. Table 4 presents the operational definitions of each motivational strategy and the studies in which each was reported.

**Table 4:** Definitions of each motivational strategy and the studies in which they were used.

Strategy	Definition	Studies that used it
Immediate feedback	Visual or auditory signals delivered during or immediately after a response	Kollins et al. (2020) [2], Jung et al. (2025) [10], Benzing and Schmidt (2019) [11], Benzing et al. (2018) [12], Zhao et al. (2024) [13], Yan et al. (2026) [14]
Adaptive difficulty	Automatic adjustment of task difficulty in real time based on performance	Kollins et al. (2020) [2], Jung et al. (2025) [10], Benzing and Schmidt (2019) [11], Zhao et al. (2024) [13], Yan et al. (2026) [14]
Points or scores	Numerical values awarded for task completion, accuracy, or speed	Kollins et al. (2020) [2], Jung et al. (2025) [10], Benzing and Schmidt (2019) [11], Benzing et al. (2018) [12]
In-game rewards	Content unlocked or earned within the intervention	Kollins et al. (2020) [2], Jung et al. (2025) [10], Yan et al. (2026) [14]
Progress tracking or levelling	Visual or structural representation of advancement	Kollins et al. (2020) [2], Jung et al. (2025) [10], Zhao et al. (2024) [13]
Token economy or virtual currency	Accumulated in-game resources exchanged for content	Jung et al. (2025) [10]
Storyline or narrative	Themed story framing the intervention tasks	Jung et al. (2025) [10]
Village-building or customisation	Player-driven modification of in-game space or characters	Jung et al. (2025) [10]
Explicit positive reinforcement	Praise, encouragement, or reward delivered after a desired response	Yan et al. (2026) [14]
Competitive leaderboards	Ranking of users against one another based on performance	None

Assessment of Executive Function and ADHD-Related Outcomes

Outcome assessment was heterogeneous. Laboratory-based or computerised measures included TOVA, Flanker, Simon, Stroop, ADS, Digit Span, and Color Span Backwards, whilst parent-rated measures included SNAP-IV, BRIEF, K-ADHD-RS, K-CPRS, and Conners-3 [2,10-14]. In-app performance was also reported in two studies [10,14].

The measures used across the six studies assess different constructs. Reaction time on an inhibition task, accuracy on a working memory task, and parent-rated inattention are not interchangeable, even when grouped under the same broad heading of executive function [28]. This distinction is relevant when interpreting the findings, because the studies do not report improvement on a single common outcome.

All six studies reported improvement in at least one outcome, although the outcomes differed between studies. Benzing et al. (2018) reported moderate improvements in inhibition and switching reaction times after a single session of exergaming, with eta-squared values of 0.117 and 0.113, respectively, but no improvement in accuracy or visual working memory [12]. Benzing and Schmidt (2019) reported moderate improvements in inhibition and switching reaction times after eight weeks of exergaming, with Cohen's d values of 0.58 and 0.65 [11]. Zhao et al. (2024) reported a large improvement in parent-rated ADHD symptoms, with a Cohen's d of 1.258 [13]. Kollins et al. (2020) reported a median change of 0.88 on the TOVA API, indicating improved objectively measured attention, but no improvement on parent-rated symptom measures [2]. Yan et al. (2026) reported a moderate improvement in attention on the TOVA API, with an eta-squared of 0.091 [14]. Jung et al. (2025) reported moderate improvements in parent-rated ADHD symptoms and behaviour problems, with Cohen's d values of 0.69 on the K-ADHD-RS and 0.72 on the K-CPRS [10].

Parent-rated outcomes appeared larger than laboratory-based measures in four of the six studies [2,10,13,14]. The included studies themselves report lack of blinding and possible expectancy effects among parents completing assessments as limitations [10,13]. Whether the observed pattern is attributable to the interventions or to the conditions under which parents rated behaviour cannot be determined from the present evidence. The effect sizes are also reported in different metrics, including Cohen's d, eta-squared, and median change. These metrics cannot be combined into a single estimate, which is consistent with the observation by Mao et al. that pooling across 20 studies produced substantial heterogeneity [6]. The variation in outcome measures and effect metrics across the six studies is therefore a notable feature of this evidence base.

Limitations of Included Studies

The included studies possessed several methodological strengths. Five of six studies employed randomised controlled designs, whilst the remaining study was a single-arm pilot study [2,10-14]. The predominance of controlled designs provides a stronger basis for examining intervention-associated changes than uncontrolled pre-post studies alone. This review did not examine non-digital interventions, such as teacher-led or parent-led strategies, dietary factors, or gut microbiome contributions, and these remain outside the scope of the current mapping.

All six studies examined children aged 6-12 years, with no study examining adolescents aged 13-18 years exclusively [2,10-14]. The findings should not be casually extrapolated to older populations. Sex representation was likewise uneven, with female participants comprising approximately 17%-31% of study populations. Only Benzing et al. (2018) reported an analysis by sex [12], and no study conducted a formal sex-based subgroup analysis. Whether these motivational strategies operate similarly across sexes remains insufficiently elucidated.

The studies also differed in ADHD diagnostic criteria, medication status, comorbidity exclusions, IQ requirements, intervention characteristics, and statistical methodology [2,10-14]. These differences may affect the comparability of the included populations and outcomes.

Limitations of This Review

The present review itself is not without limitations. Most notably, as shown in Figure 2, the search was conducted in PubMed alone. Although PubMed constitutes a substantial biomedical database, reliance upon a single database may have resulted in relevant literature indexed elsewhere being omitted. Restriction to English-language publications and freely available full-text articles may have further narrowed the evidence identified.

Only six met the predefined inclusion criteria. This number follows from the narrow scope of the review and from the limited number of primary studies that explicitly describe motivational strategies in this population.

Heterogeneity was another principal limitation. The included studies differed in intervention format, duration, session structure, motivational strategies, outcome measures, and statistical methodology. This precluded meaningful quantitative pooling and made descriptive synthesis more appropriate. The purpose of a scoping review is not necessarily to force disparate evidence into a singular numerical estimate, but rather to map what evidence exists and where the gaps remain [7].

The principal strengths of this review lie in its use of the PRISMA-ScR framework, independent screening by two reviewers, critical appraisal using the JBI checklists, and a search conducted in PubMed. The review provides a systematic mapping of motivational strategies across digital interventions for children with ADHD and draws attention to the gap between the presence of motivational design features and the absence of evidence about which features matter.

Implications for Stakeholders and Future Directions

The findings have implications for intervention developers, researchers, clinicians, and families. For intervention developers, the review provides a preliminary catalogue of motivational strategies already incorporated into digital interventions for children with ADHD. Immediate feedback and adaptive difficulty appear particularly prevalent, whilst virtual currency, narrative, and customisation have been less frequently reported.

For clinicians and families, however, these findings should not be interpreted as establishing that one motivational strategy is preferable to another. The evidence does not presently permit such a conclusion. Rather, it suggests that motivational design has become increasingly intertwined with digital intervention design itself.

Future studies should describe motivational components with substantially greater specificity. Merely stating that an intervention was “gamified” is insufficient to elucidate what the child actually encountered. Researchers should identify individual strategies, describe their implementation, and, where possible, report engagement alongside cognitive and ADHD-related outcomes.

There is also a need to distinguish the contributions of individual components. Component-based or factorial designs may prove useful in determining whether feedback, rewards, adaptive difficulty, progression, or other features contribute independently to engagement or treatment response. Greater diversity in recruitment, greater representation of female participants and adolescents, and longer follow-up should likewise be prioritised.

## Conclusions

This review set out to examine what motivational strategies are actually embedded within digital interventions for children with ADHD. Six studies met the inclusion criteria. Each incorporated at least one strategy. Immediate feedback was present in all six, and adaptive difficulty in five. Points or scores, rewards, and progress tracking appeared in fewer studies. Narrative elements, virtual currency, customisation, and explicit positive reinforcement each appeared once. Competitive leaderboards appeared in none. The difficulty is that we cannot say which of these strategies mattered. Each study combined several strategies within the same intervention, which makes their individual contributions impossible to isolate. A child who improved may have improved because of the feedback, the rewards, the cognitive training itself, the duration of the intervention, or something not measured. The strategies were present. Their effects remain unclear.

Future work would benefit from treating motivational strategies as separate components rather than as a single design feature, and from measuring outcomes in ways that allow comparison across studies. Whether these strategies sustain a child's engagement beyond the initial weeks of an intervention is still unknown. The field has assembled a preliminary catalogue of motivational tools. It does not yet have a clear account of how they function.
